# XPD–The Lynchpin of NER: Molecule, Gene, Polymorphisms, and Role in Colorectal Carcinogenesis

**DOI:** 10.3389/fmolb.2018.00023

**Published:** 2018-03-20

**Authors:** Aga Syed Sameer, Saniya Nissar

**Affiliations:** ^1^Department of Basic Medical Sciences, College of Medicine, King Saud bin Abdulaziz University for Health Sciences, Jeddah, Saudi Arabia; ^2^Department of Biochemistry, Kashmir University, Srinagar, India

**Keywords:** colorectal cancer, polymorphism, RFLP, DNA repair, NER, XPD, TFIIH, xeroderma pigmentosum

## Abstract

In mammals the bulky DNA adduct lesions known to result in deleterious phenotypes are acted upon and removed from the genomic DNA by nucleotide excision repair (NER) pathway. TFIIH multi-protein complex with its important helicase–Xeroderma Pigmentosum Protein (XPD) serves as the pivotal factor for opening up of the damaged lesion DNA site and carry out the repair process. The initial damage verification step of the TFIIH is in part dependent upon the helicase activity of XPD. Besides, XPD is also actively involved in the initiation steps of transcription and in the regulation of the cell cycle and apoptosis. In this review, we will be exploring the new insights in scientific research on the functioning of the NER pathway, the role of TFIIH as the central complex of NER, the pivotal helicase XPD as the lynchpin of NER and the effects of various single nucleotide polymorphisms (SNPs) of XPD on its functioning and their consequent role in colorectal carcinogenesis.

## Introduction

Colorectal carcinogenesis is a multifactorial and multigene process that is controlled by various gatekeeper and caretaker genes via definite pathway, referred to as the adenoma–carcinoma sequence/model (Vogelstein et al., 1988). The development of colorectal cancer (CRC) has been shown to occur because of cumulative accumulation of mutations in tumor-suppressor genes and oncogenes. The process of transformationinvolves several genetic changes that are essential for cancer initiation and progression (Migliore et al., 2011). On the basis of molecular profiles, CRC is now classified into three specific phenotypes (Cunningham et al., 2010); involving three major genetic instability pathways: chromosomal instability (CIN), microsatellite instability (MSI) and CpG island methylator phenotype (CIMP) (Ogino et al., 2011; Sameer, 2013).

The CIN pathway is the major mediator of colorectal tumorigenesis and is responsible for approximately 80–85% of all colorectal cancers (Goel et al., 2003; Grady, 2004). The CIN pathway involves the dynamic and continued loss or gain of whole chromosomes or chromosomal regions (due to sub-chromosomal genomic deletion and amplification respectively) that carry genes of critical importance to the process of colorectal tumorigenesis. The major initiating events in the CIN pathway are the induced mutations in two classes of critical gate-keeper genes, oncogenes and tumor suppressor genes (McGranahan et al., 2012), which in turn lead to the dysregulation of several critical signaling pathways that characterize the CIN pathway.

Microsatellite instability (MSI) usually comprises the length alterations of oligonucleotide repeat sequences (microsatellites) that occur somatically in many human tumors. The microsatellites are highly prone to replication errors due to higher susceptibility of DNA polymerase to induce mismatches during replication of long or short repetitive DNA sequences including MSs and the mutations in the form of insertions or deletions often termed as an insertion-deletion loop leads to length alterations and result in MSI. Mutations in DNA mismatch repair (MMR) genes result in a failure to repair errors that occur during DNA replication in microsatellites, resulting in an accumulation of frameshift mutations in genes that contain microsatellites (Boland and Goel, 2010). The MMR system in humans consists of several identified proteins, MLH1, MLH3, MSH2, MSH3, MSH6, PMS1, PMS2, and Exo1 with MLH1 and MSH2 being the important members (Sameer et al., 2014). MSI is also found in 12–15% of sporadic CRCs. MIN tumors are more frequently right-sided and poorly differentiated, and more often display unusual histological type (mucinous), and marked peri-tumoral and intra-tumoral lymphocytic infiltration (Benatti et al., 2005). MSI also occurs in patients with ulcerative colitis and is fairly common in premalignant (dysplastic) and malignant lesions (21 and 19% respectively; Kerr et al., 2001).

CpG island methylator phenotype (CIMP), also referred to as methylator phenotype of CRC tumorigenesis involves gene silencing or transcriptional inactivation by CpG island methylation in tumor suppressor gene promoters. It plays a major role in about 35% of CRCs (Sporadic as well as Hereditary) (Cunningham et al., 2010; Sameer and Nissar, 2016). Thus, under epigenetic point of view, classically CRC has been divided essentially into CIMP positive (CIMP+) and non-CIMP (CIMP negative) tumors (Toyota et al., 1999). The genes implicated in colorectal tumorigenesis that are silenced by hypermethylation include DNA repair genes such as methylguanine methyltransferase (MGMT) and MLH1, tumor suppressors (p16, APC, insulin-like growth factor 2, and HIC1), cell cycle regulatory genes (Mutated In Colorectal Cancers/MCC) and Wnt signaling antagonists known as SFRPs (secreted frizzled-related proteins). The silencing of MLH1 gene by hypermethylation is a frequent event in sporadic MSI-High colorectal tumors (Kim et al., 2010).

One of the main causes of genetic instability leading to tumorigenesis is the inefficient repair of DNA lesions that creep in the cellular genome via various DNA damaging agents affecting the normal cellular functioning.

## DNA repair mechanisms

The eukaryotic genome is always under a constant threat of wide variety of DNA-damaging agents (physical & chemical) which affect the structure and function of the genes. To prevent the effect of DNA modifying agents on the functionality of the genome and hence to allow the smooth functioning of the fundamental cellular processes, cells have a varied arsenal of a number of repair molecules and pathways that are specifically used to identify and remove the several specific types of DNA lesions which may otherwise lead to various diseases primary among them-cancers (Michailidi et al., 2012). However, each of the DNA repair pathway is specific to the particular type of DNA lesion. Hence the mechanism of restoration of intact DNA depends on various factors like the cell cycle phase, type of lesion and other environmental factors. But, one thing that is common to all repair mechanisms is that all of them use the cell's own intact complementary DNA strand as a template to restore the original strand. The various repair mechanisms operating within the confines of the cell can be divided broadly into two types: direct and indirect; depending upon the molecules/proteins used for the repair process and also the time period of the repair mechanisms (Michailidi et al., 2012; Sameer et al., 2014).

Direct repair mechanisms are carried out during the replication process itself, when the daughter DNA is being synthesized within the cell. These mechanism are mostly mediated by direct DNA interacting enzymes which may be either DNA polymerases themselves or O6-methylguanine DNA methyltransferase (MGMT) (Hoeijmakers, 2001; Michailidi et al., 2012; Iyama and Wilson, 2013; Sameer et al., 2014). Indirect repair is carried out after the synthesis process and mostly involves the repair of those lesions that occurred during the synthesis of DNA. Being essentially a post-replication process it serves to the inability of the direct repair to fix all lesions during the synthesis process (Sameer et al., 2014). Thus, essentially, it is a post synthesis process of correcting the DNA lesions, and is usually assisted by various proteins which are involved in DNA replication as well.

Indirect DNA repair mechanisms are further classified into three categories: excision repair (ER), recombination repair (RR), and mismatch repair (MMR). ER has two subcategories, namely, base excision repair (BER) for excision of abnormal bases such as uracil and breaks found only in one DNA strand, and nucleotide excision repair (NER) for the removal of bulky adducts (Michailidi et al., 2012; Iyama and Wilson, 2013; Sameer et al., 2014).

Recombination repair (RR), also called as homologous recombination (HR) comprises a series of interrelated pathways that function in the repair of DNA double-stranded breaks (DSBs) and interstrand crosslinks (ICLs) using a homologous DNA (Jasin and Rodney, 2013). MMR repairs the small loops within the duplex DNA that arise from nucleotide misincorporations—either by base—base mismatches or by insertion/deletion loops (Sameer et al., 2014).

## NER: structure and function

The NER system is one of the principle mechanisms that cells employ in protecting themselves against genotoxic damage such as that induced by UV-irradiation or exposure to chemical carcinogens which are known to incorporate DNA lesions in the cellular genome (Benhamou and Sarasin, 2000, 2002). The most common DNA lesions which serve as the substrates of NER system are bulky covalent adducts in DNA double strands. They are produced from nitrogenous bases of DNA when exposed to strong mutagens like UV light, ionizing radiations, electrophilic chemical mutagens, drugs, and chemically active endogenous metabolites (Petruseva et al., 2014). The inability of BER to remove the bulky lesions from the duplex DNA is dealt upon by NER. In general, NER corrects the helix-distorting base lesions within the DNA that arise because of exposure to sunlight or bulky chemicals such as benzo[a]pyrene, benzo[c]anthracene, diol-epoxide, aromatic amines such as acetyl-aminofluorene, aflatoxin, nitrosamines such as MNNG, and 4-nitroquinoline oxide (Nouspikel, 2009; Michailidi et al., 2012; Petruseva et al., 2014).

The NER pathway repairs the damaged strand by excising about 24–32nt DNA fragments containing the DNA lesion (Petruseva et al., 2014). The repair of the damaged DNA strand involves five main steps; it begins with the damage recognition step, opening of the double helix at the lesion site, demarcation of the actual DNA lesion and assembly of a pre-incision complex, followed by excision of the lesion containing damaged strand and synthesis of the DNA in the gap (Benhamou and Sarasin, 2000, 2002; Hoeijmakers, 2001). Each of these steps requires the functioning of the specialized protein complexes to carry out the repair efficiently and specifically (Spivak, 2015). Till date about 30 different polypeptides have been identified which play a pivotal role in one or more steps of the NER pathway (Petruseva et al., 2014).

Based upon the initial damage recognition mechanisms, NER pathway has two functionally distinct branches for dealing with DNA lesion which distort its helical structure. One operates to repair bulky lesions throughout the entire genome (i.e., global genome NER, GG-NER) including the untranscribed regions and silent chromatin. The second works in cooperation with the transcription machinery to remove damage from actively transcribed regions of genes (i.e., transcription-coupled NER, TC-NER) (Petruseva et al., 2014; Spivak, 2015).

GG-NER is controlled by the specialized protein called XPC (Xeroderma Pigmentosum C) which senses and recognizes the helix distorting DNA lesion in the genome to start the repair process. However, some lesions like cyclobutane pyrimidine dimers (CPD) which are too small to destabilize the DNA helical structure are recognized first by damage-specific DNA binding protein 1 (DDB1) and DDB2/XPE complex (Spivak, 2015). XPC is functional as a hetero-trimer in complex with two other proteins - human RAD23 homolog B (hRAD23B /HR23B) and centrin 2 (CETN2). HR23B stabilizes the complex, protects it against proteasome degradation, and stimulates the DNA-binding activity of XPC. It plays a pivotal role in the recruitment of other repair proteins into the GG-NER process (Nouspikel, 2009; Petruseva et al., 2014; Spivak, 2015).

TC-NER is contrarily dependent upon the transcription machinery for its initial recognition of the DNA lesion. In TC-NER, DNA damage is detected by the elongating RNA polymerase II (RNAPII) when it encounters a bulky DNA lesion within the coding region of the gene to be transcribed (de Laat et al., 1999). So the blocking of the RNAPII by the damaged DNA constitutes the first step for the damage repair via TC-NER (Spivak, 2015). The arrested elongation complex then recruits CSB (ERCC6), a transcription elongation factor that translocates along template DNA with RNAPII. CSB acts as a master recruiter then by recruiting complexes of proteins required for repair mechanism like the CSA complex, NER factors (not including the GGR recognition factors XPC and XPE) and p300 to sites of arrested RNAPII. Both branches of the NER–TC-NER and GG-NER then converge with the recruitment of transcription factor II H (TFIIH) to the repair site (Spivak, 2015; Table 1).

**Table 1:** Composition of the human NER system.

**Factor**	**Subunits**	**Function**	**Additional role**	**Interactions with**
XPC	HR23B	Stimulates XPC activity	Protects XPC complex from proteasome degradation	TFIIH; XPA; DDB
	XPC	Recognition of a distorted DNA lesion	Works in GG-NER only	
	CEN2	Stabilize the binding of XPC to DNA lesion	Regulates recruitment of TFIIH	
DDB	DDB1	Recognition of damage, interaction with chromatin		XPA; RPA
	DDB2			
XPA	XPA	Structural function, binding to a damaged strand and facilitating repair complex assembly		XPA; RPA; TFIIH; ERCC1
RPA	RPA70	Stabilizes single-stranded DNA and positions nucleases	Replication and Recombination	XPA; XPG; PCNA/RFC
	RPA32			
	RPA14			
XPF	ERCC1	Endonuclease, catalyzes formation of single-strand break in DNA on the 5′ side of the damage	Interstrand cross-link repair	XPA; TFIIH
	XPF		Recombination via single-strand annealing	
XPG	XPG	Endonuclease, catalyzes formation of single-strand break in DNA on the 3′ side of the damage	Member of FEN-1 family of nucleases	TFIIH; PCNA; RPA
RFC	RFC1	ATP-dependent connection of PCN A		PCNA; RPA
	RFC2			
	RFC3			
	RFC4			
	RFC5			
PCNA	PCNA	Factor ensuring processivity of DNA polymerases		RFC; XPG; Pol δ
Pol δ	pI25	DNA polymerase		PCNA
	p66			
	p50			
	p12			
Pol ε	p261	DNA polymerase		PCNA
	p59			
	p17			
	p12			
Ligase I	Ligase I	Ligation of a single-strand break		
Ligase III	Ligase III			
TFIIH		Discussed in Table 2		XPA; XPC; XPF; XPG

*Adapted from Petruseva et al. (2014)*.

Defects in NER usually results in UV-sensitive and high-carcinogenis pathologies; and have been shown to cause at least three human genetic disorders: xeroderma pigmentosum (XP), Cockayne's syndrome (CS), and trichothiodystrophy (TTD); in addition to neurodegenerative manifestations (Iyama and Wilson, 2013; Petruseva et al., 2014; Spivak, 2015).

## TFIIH: structure and function

TFIIH is a remarkable dual function multisubunit protein complex that not only plays a fundamental role in the transcription of protein-coding genes but also plays an important role in the NER system (Oksenych and Coin, 2010; Compe and Egly, 2012). In transcription, TFIIH is essentially required for two purposes - first for the proper binding of the RNA polymerase I & II at their specific promoter regions just upstream of the gene and second for the promoter clearance of polymerase to proceed into elongation phase of transcription via its C-terminal domain (CTD) phosphorylation (Mydlikova et al., 2010; Compe and Egly, 2012). In TC-NER, TFIIH forms a part of the core incision machinery without which NER system would not function (Egly and Coin, 2011).

**Figure 1:**
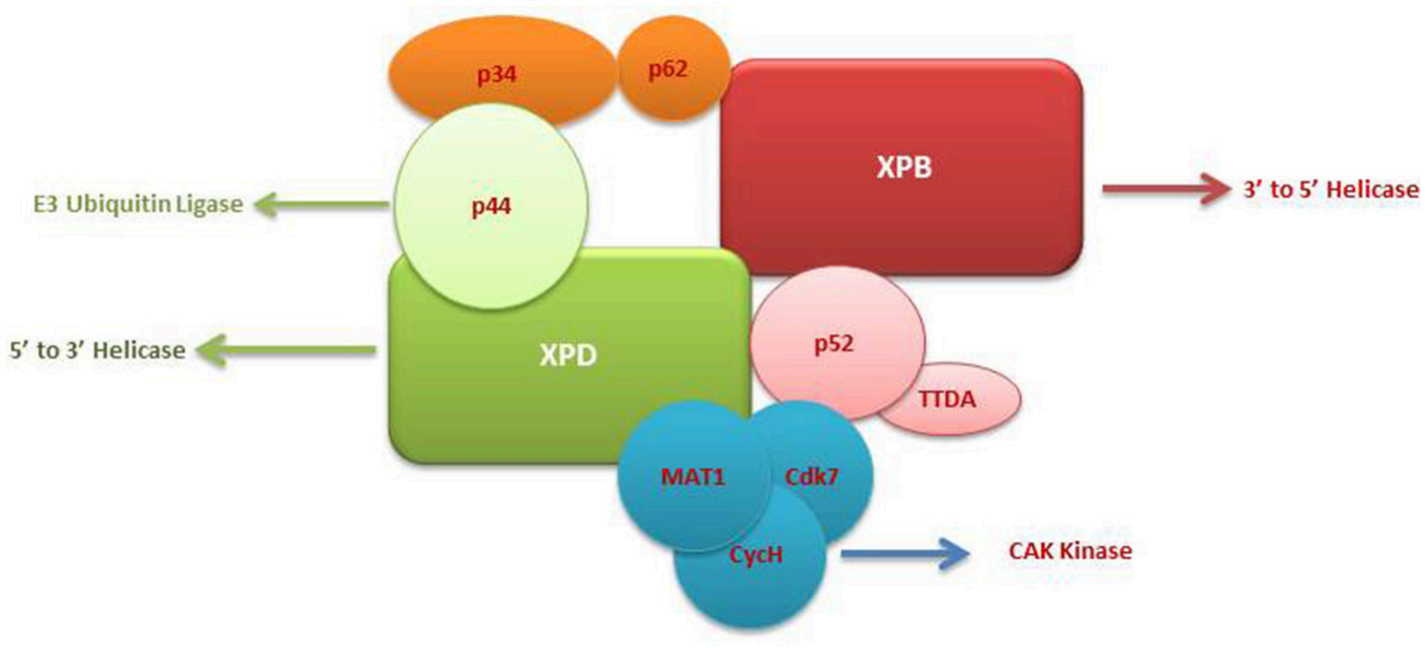
A multisubunit functional complex of TFIIH. The TFIIH complex is composed of eleven subunits, with XPB making its one face and XPD another. The complex also contains CAK kinase domain of three subunits (Blue). Functonally TFIIH possess four enzymatic activities; XPB and XPD has helicase activity, Cdk7 has kinase and p44 serves as E3 ubiquitin ligase.

The TFIIH complex is composed of two sub-complexes: core complex and cyclin-dependent kinase (CDK)-activating kinase (CAK) complex. The 3D structure of TFIIH is organized into ring-like core from which the CAK module protrudes out (Chang and Kornberg, 2000). Core complex consists of seven subunit core (XPB, p62, p52, p44, p34, and TTD-A) associated with a three subunit CAK module by the XPD helicase (Oksenych and Coin, 2010; Table 2, Figure 1). CAK module is composed of CDK7 (p40), cyclin H (p34) and menage á trois 1 (MAT1; p32). Three important enzymatic subunits are present within the confines of TFIIH complex, two ATP-dependent DNA helicases: XPB and XPD, and the kinase CDK7 (Mydlikova et al., 2010; Zhovmer et al., 2010). Proteins p62, p52, p44 and p34 previously regarded just as “structural” subunits have been shown to contain regulatory functions within TFIIH. Protein p52 modulates XPB activity by upregulating its ATPase activity through a direct XPB/p52 interaction and also anchors XPB to the TFIIH; while as protein p44 regulates XPD via direct p44/XPD interaction and also functions as ubiquitin ligase and protein p62 has been demonstrated to interact with thyroid hormone receptor TRβ (Mydlikova et al., 2010; Egly and Coin, 2011; Compe and Egly, 2012).

**Table 2:** Composition of the human TFIIH complex.

	**Human**	**Yeast**	**Function**	**Related human disorders**
Core complex	XPB	Ssl2	3′-5′ ATP-dependent helicase	Trichothiodystrophy and combined xeroderma pigmentosum and Cockayne syndrome
	XPD	Rad3	5′-3′ ATP-dependent helicase and forms a bridge between the CAK and the core	Trichothiodystrophy, xeroderma pigmentosum and combined xeroderma pigmentosum and Cockayne syndrome
	P62	Tfb1	Structural function and interacts with transcription factors and NER factors, stimulates XPB	
	P52	Tfb2	Regulates the XBP ATPase activity	
	P44	Ssl1	E3 ubiquitin ligase^*^, stimulates XPD	
	P34	Tfb4	Structural function and strong interaction with p44	
	P8	Tfb5	Interaction with P52, stimulation of XPB ATPase activity	Trichothiodystrophy
CAK module	CDK7	Kin28	Kinase	
	Cyclin H	Ccl1	Modulates the CDK7 kinase activity	
	MAT 1	Tfb3	CAK stabilization and regulates cullin neddylation^*^	

*CAK, cyclin-dependent kinase-activating kinase subcomplex; Ccl1, cyclin C like 1; CDK7, cyclin-dependent kinase 7; MAT1, ménage à trois 1; NER, nucleotide excision repair; Ssl, suppressor of stem–loop protein; Tfb, RNA polymerase II transcription factor b; XPB, xeroderma pigmentosum group B complementing protein*.

* *Activity found in yeast only*.

The isolated XPB and XPD subunits of TFIIH are both ATP-dependent DNA helicases with 3′ → 5′ and 5′ → 3′ DBA helicase activity respectively (Mydlikova et al., 2010). XPB and XPD with opposite polarities have been suggested to cooperate in the opening of the damaged DNA helix on opposite sides of a lesion, with XPB working on the 3′ side of the lesion and XPD working from the 5′side (Oksenych and Coin, 2010; Fuss and Tainer, 2011; Compe and Egly, 2012). However, the differential role play by both proteins has been shown to toggle between transcription functions and NER functions (Oksenych and Coin, 2010); wherein XPB ATPase activity is essentially required for DNA opening in both NER and transcription but its helicase activity is dedicated to only promoter escape in transcription process. In contrast, helicase activity of XPD plays a minor role in transcription process but it is pivotal in NER system for the removal of DNA lesions (Tirode et al., 1999; Winkler et al., 2000; Coin et al., 2007; Richards et al., 2008; Oksenych and Coin, 2010). The opening of the DNA helix around the lesion by these two helicases in an ATP-dependents manner is the first catalytic reaction of NER system which in turn leads to the conformational changes that allows the recruitment of additional NER proteins (Wolski et al., 2010).

Within the core complex of TFIIH XPB constitutes the biggest subunit containing seven helicase motifs and belongs to helicase superfamily 2 (SF2); while as XPD contains a RecA-like fold that belongs to the SF2 family of helicases with distinct 4Fe4S (FeS) cluster that is essential for its function as a helicase differentiating into two helicase domains, HD1 and HD2 (Zurita and Merino, 2003; Rudolf et al., 2006; Mydlikova et al., 2010; Wolski et al., 2010).

In CAK domain, CDK7 protein is the biggest subunit with bifunctional activity - one phosphorylase via which CDKs participate in the cell cycle, and second as a component of the TFIIH, which is essential for CTD phosphorylation of the largest subunit of RNA polymerase II (Mydlikova et al., 2010). MAT1 protein functions to link CAK to the core TFIIH in a complex interaction which is facilitated also by both XPD and XPB helicases. MAT1 interacts with the CDK7-cyclin H complex and stimulates the CDK7 kinase activity (Busso et al., 2000).

TFIIH role in transcription is via its joining the other general transcription factors like TFIIA, TFIIB, TFIID, TFIIE, and TFIIF to form the preinitiation complex (of more than 30 polypeptides) together with central RNPII at the promoter region of the gene to be transcribed. Promoter recognition is initially carried out by TFIID which in turn recruits TFIIA and TFIIB eventually TFIIH entry is mediated via TFIIF/E (Mydlikova et al., 2010; Compe and Egly, 2012). In transcriptional process, TFIIH plays wide variety of roles; it is involved in initiation, promoter escape, and early elongation stages, to transcription reinitiation and formation of gene loops (Zhovmer et al., 2010). TFIIH controls the initiation of transcription and enhances the association of the RNPII CTD with the 7-methylguanosine (m7G) RNA capping machinery (Serizawa et al., 1993). This TFIIH kinase activity toward the CTD of Pol II can be modulated by different factors, including MAT1 (ménage à trois 1) and cyclin H, which are two binding partners of CDK7 within the CAK subcomplex (Komarnitsky et al., 2000). It is also plays important role in the RNPI transcription of ribosomal genes (Iben et al., 2002).

**Figure 2:**
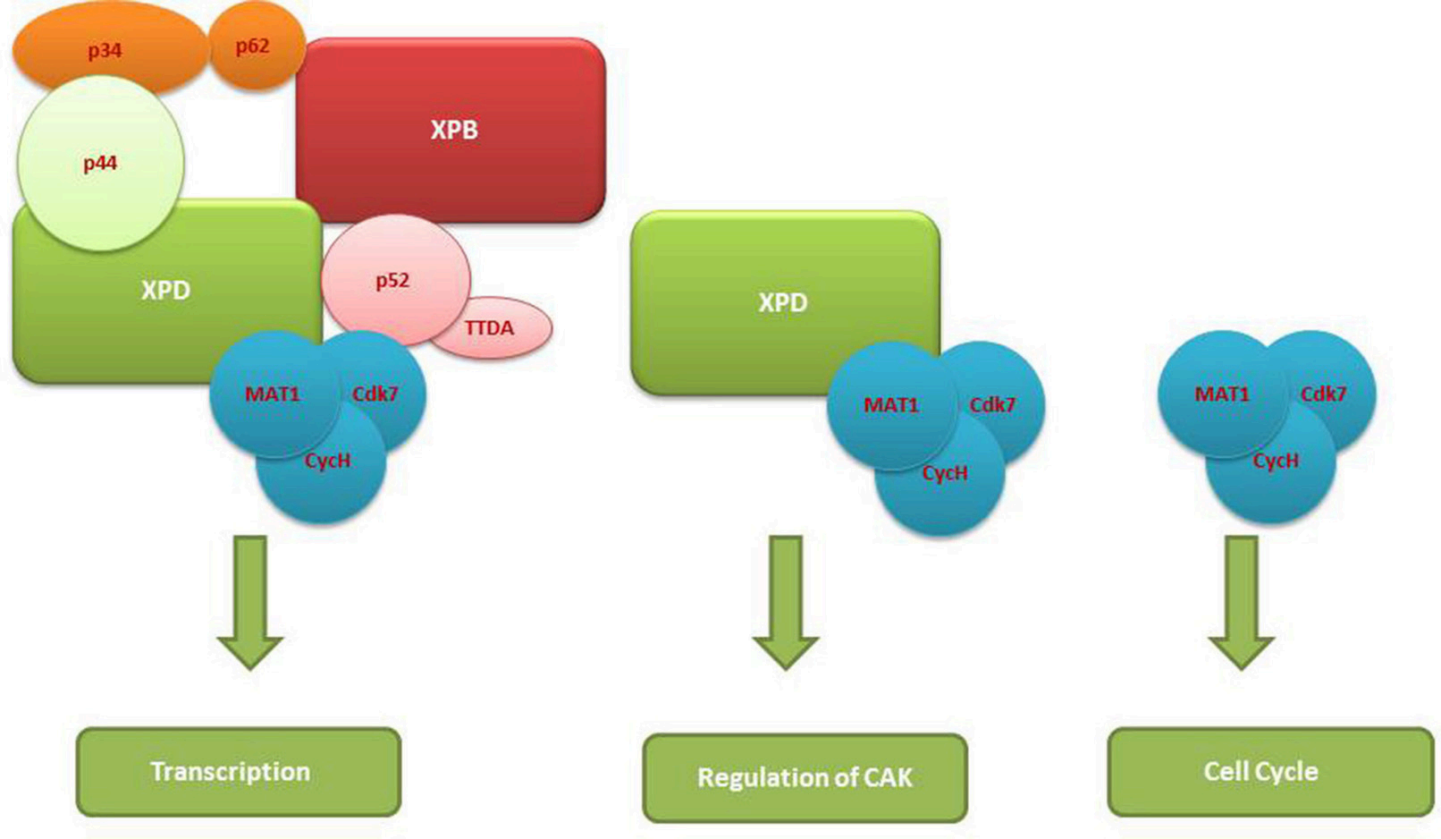
TFIIH complexes vs. non-TFIIH complexes and differences in their functions.

As already mentioned, depending upon the branches of the NER whether GG-NER or TC-NER, the recruitment of TFIIH is mediated by either XPC or by the stalled RNPII to the site of the lesion in the DNA. After recruitment, it functions to open the DNA around the lesion and thereby allow the excision of the string of 24–32nt DNA fragments containing the lesion and its consequent replacement by a new DNA fragment (Oksenych and Coin, 2010; Egly and Coin, 2011; Compe and Egly, 2012). In GG-NER, TFIIH is a part of the dual incision complex composed of XPC-HR23B, centrin2, XPA, replication protein A (RPA), XPG, and excision repair cross-complementation group 1 (ERCC1)-XPF, and is involved in the opening of the DNA around the lesion (Figure 2). Because of its high sensitivity for the recognition of damages sites; XPC not only rapidly detects the various DNA lesions but it also promotes the kinks in DNA helix forming a transient recognition intermediate; allowing the other proteins of NER to be recruited to the site (Compe and Egly, 2012). XPA is known as a scaffold protein without enzymatic activity that nevertheless shows preferential association to damaged DNA and is indispensable for DNA incision (Missura et al., 2001). After correctly seated at the damaged DNA, TFIIH then mediates the excision of the DNA lesion with the help of XPB and XPD ATPase/helicase activities (Oksenych et al., 2009).

## XPD: structure and function

XPD (also known as ERCC2) is a helicase protein of 761 amino acids with a molecular weight of 86.9kDa (Benhamou and Sarasin, 2002). XPD is one of the two pivotal ATPase/Helicase of the core unit of THIIF molecular assembly. It seems to form a bridge between the TFIIH core complex and the CAK module–which otherwise also exists as a free trimeric complex with its own distinct functions (Chen and Suter, 2003; Cameroni et al., 2010). XPD belongs to an ATP-dependent 5′-3′ superfamily 2 (SF2) helicases, which are characterized by seven “helicase motifs” (walker motif I, Ia, II, III, IV, V, and VI) constituted of highly conserved amino-acid sequences (Oksenych and Coin, 2010). Interestingly, the XPD protein also constitutes a 4Fe4S (FeS) cluster that has been demonstrated to be essential for its helicase activity. Because of this cluster XPD becomes a founding member of a family of related SF2 helicases (Rudolf et al., 2006, 2010; Liu et al., 2008). SF2 family helicases also comprises various important family members like bacterial DinG (damage-inducible G) and the eukaryotic XPD paralogs FancJ (Fanconi's anemia complementation group J), RTEL (regular of telomere length) and Chl1 (chromatid cohesion in yeast) (Wolski et al., 2010). The exact function of the FeS cluster is not known but a number of explanations as to its role have been given like–a purely structural role and providing stabilization to the FeS domain; direct interaction with the damaged DNA substrate and acting as a damage sensor and acting as a regulatory center for XPD helicase (Rudolf et al., 2006; Fan et al., 2008; Wolski et al., 2008, 2010; Houten et al., 2016).

Furthermore, XPD serves as the authenticator of the DNA lesion initially sensed by XPC-HR23B which preludes the binding of TFIIH at the site of lesion (Oksenych and Coin, 2010). The opening of the DNA duplex at the site of lesion requires the dual ATPase function of both XPB and XPD, but the helicase activity of XPD plays a critical role in the opening of the DNA. The biochemical data vividly demonstrated that mutations in the motif I (containing ATPase activity) of either XPB or XPD inhibits the formation of DNA bubble at the lesion site but the mutations in the motif III and IV (containing helicase activities) of XPB impairs its functionality but does not inhibit NER *in vivo* (Coin et al., 2007). However, some specific mutations in both XPB and XPD can completely prevent opening and dual incision of the DNA lesions site in NER (Evans et al., 1997). Additionally, it has also been demonstrated that binding of N-terminal p44 subunit with XPD stimulates its helicase activity by almost 10-fold. Furthermore, mutations in the C-terminal domain (CTD) of XPD prevents the interactions with p44 resulting not only in decrease in the overall TFIIH helicase activity but also modulates TFIIH composition and contributing to further transcription defects (Coin et al., 1998, 1999). XPD has also been demonstrated to control the cell cycle via its interaction with CAK domain of the TFIIH complex. Downregulation of XPD as happens at the beginning of the mitoses initiates the disengagement of CAK module from TFIIH complex and its eventual role as regulator of cell cycle independent of TFIIH core complex (Chen et al., 2003).

## XPD gene SNPs, DNA repair capacity and CRC

XPD gene is located at chromosome 19q13.3 and comprises of 23 exons which span around ~54.3 kb in length; cDNA of this gene is about 2,400 nt (Benhamou and Sarasin, 2002). Point mutations in the human XPD protein play a causative role in DNA repair-deficiency diseases (xeroderma pigmentosum, trichothiodystrophy, and Cockayne syndrome), which are characterized by high ultraviolet-light hypersensitivity, a high mutation frequency, and cancer-proneness, as well as some mental and growth retardation and probably aging. Till date almost 100 different mutations have been in the XPD gene (Itin et al., 2001; Fan et al., 2008) (most important are given in Table 3). Most of the mutations affecting XPD are clustered in the C-terminal domain of the protein, which is the pivotal interaction domain of XPD for p44, as already pointed out in the above section (Coin et al., 1998, 1999; Fan et al., 2008; Liu et al., 2008; Wolski et al., 2008, 2010).

**Table 3:** Most common mutations affecting XPD protein.

**S. No**	**Human**	**Sulfolobus acidocaldarius**	**Motif affected**	**Disease**
1	T76A	T56A	Ia	XP
2	D234N	D180N	II	XP
3	Y542C	Y403C	IV	XP
4	R601L/W	K446L	V	XP
5	R638W/Q	R531W		XP
6	K507Q	K369Q	Channel	
7	G47R	G34R		XP/CS
8	G602D	G447D		XP/CS
9	R666W	R514W	VI	XP/CS
10	G675R	C523R		XP/CS
11	R112H	K84H		TTD
12	R592P	K438P	V	TTD
13	D673G	D521G		TTD
14	C116	C88S	4Fe-4S	
15	C134	C102S	4Fe-4S	

*Adapted from Fan et al. (2008)*.

In addition to the deleterious point mutations, a number of single nucleotide polymorphisms (SNPs) have been found in the *XPD* gene affecting both exonic and intronic regions of the gene). Till date researchers have defined 17 different SNPs in the XPG gene in his seminal study; seven of which affected the coding regions of the gene (exons 6, 8, 10, 17, 22, and 23) and hence affected XPD enzymatic activity (Shen et al., 1998; Mohrenweiser et al., 2002). Among all, four SNPs result in amino acid changes: isoleucine to methionine in codon 199 (C > G), histidine to tyrosine in codon 201 (C > T), aspartic acid to asparagine in codon 312 (G > A) and lysine to glutamine in codon 751 (A > C). Out of these four; only two are the most commonly occurring ones—codon 312 and 751 while as other two—codon 199 and 201 are rare (Shen et al., 1998; Benhamou and Sarasin, 2002; Gdowicz-Klosok et al., 2013).

Among all the reported SNPs of XPD, most of the population based case control studies have focused on studying the effects of SNPs affecting codons 156, 312, and 751 only, partly because of their high occurring frequency and partly because of their effects on XPD helicase activity (Coin et al., 1998; Shen et al., 1998; Benhamou and Sarasin, 2002).

XPD Asp312Asn and Lys751Gln two of the most common SNPs located within the exon 23 of the *XPD* gene which affects the C-terminal domain of XPD helicase that is known to interact with p44 protein of TFIIH complex, thereby stimulating XPD helicase activity (Coin et al., 1999). Thus, these two SNPs may therefore affect different protein interactions; diminish the activity of TFIIH complexes (Shen et al., 1998). In addition, XPD Lys751Gln SNP is also known to reduce the XPD protein expression by decreasing the mRNA stability (Moisan et al., 2012).

Lunn et al. (2000) was the first to report the reduced repair of X-ray induced DNA damage by XPD Lys751Lys genotype. It was reported that Individuals with the XPD 751 Lys/Lys genotype had a higher number of chromatid aberrations than those having a 751Gln allele. Possessing a Lys/Lys751 genotype increased the risk of sub-optimal DNA repair by almost 7 folds, suggesting that the Lys751 (common) allele may alter the XPD protein product resulting in sub-optimal repair of X-ray-induced DNA damage.

Furthermore, it has been also reported that the XPD Lys751 allele is associated with a high level of UVC-induced formation of DNA strand breaks (Møller et al., 2000). Also, Lunn et al. (2000) suggested that XPD Lys751 may alter the XPD protein product resulting in the suboptimal repair of X-ray induced DNA damage. However in contrast, two studies reported that the cells containing the homozygous Lys/Lys XPD protein had the elevated repair capacity than the cell containing XPD protein with Gln in either of the two forms (Spitz et al., 2001; Qiao et al., 2002).

A large number of epidemiological studies have been carried out recently to understand the effects and role of XPD SNPs on the modulation of risk of CRC; while some studies found a significant association between the two (Skjelbred et al., 2006; Gan et al., 2012; Huang et al., 2013; Procopciuc and Osian, 2013; Rezaei et al., 2013; Paszkowska-Szczur et al., 2015) others failed to link them (Yeh et al., 2005; Zhang et al., 2011, 2014; Du et al., 2014; Moghtit et al., 2014).

Two important recent meta-analyses - one by Zhang et al. (2014) on 11 case-control studies (including a total of 3 2,961 cases and 4,539 controls) and another by Zhang et al. (2011) on 15 case–control studies (including a total of 3,042 cases and 4,627 controls) did not found any evidence of a link between the XPD Lys751Gln polymorphism and risk of CRC. A recent study by Moghtit et al. (2014) on Western Algerian CRC patients (consisting of 129 cases and 148 controls) reported no association of the XPD Lys751Gln with CRC risk. Furthermore, Sliwinski et al. (2009) in their study on polish CRC cases did not find any significant association between any genotype of XPD 751 codon SNP and the occurrence of CRC; they also did not observe any relationship between XPD 751 SNP and any of the clinicopathological parameters.

Paszkowska-Szczur et al. (2015) in their study on polish CRC patients observed a significant association of XPD 312 SNP with the risk of developing CRC and strongly in men. Also, the study of Rezaei et al. (2013) in their study on Iranian CRC cases observed that individuals with heterozygous variant (Lys/Gln) SNP of XPD gene may have an increased susceptibility to CRC compared to other SNPs (Lys/Lys and Gln/Gln). Furthermore, they observed that heterozygous variant (Lys/Gln) was more frequent in CRC patients than in the control group. Similar results were also reported previously by Skjelbred et al. (2006) and Moreno et al. (2006) in their own respective populations. Also, Stern et al. (2006) have demonstrated lower risk of developing CRC in homozygous (Lys/Lys) SNP carriers. Contrarily, the study of Wang et al. (2010) on Indian CRC patients found that XPD 751Gln allele demonstrated the 3.5 times increased risk of rectal cancer.

However, meta-analysis by Mandal et al. (2014) of 13 case-control studies (including 3,087 cases and 3,599 controls) reported the likely association of the XPD Lys751Gln polymorphism with the risk of development of cancer in Indian population. Their meta-analysis concluded that XPD Lys/Gln and XPD Gln/Gln genotypes had had 1.3- and 1.6-fold increased risk of developing cancer as compared with the wild XPD Lys/Lys genotype, respectively. Similarly, another meta-analysis of 37 case-control studies (including 9,027 cases and 16,072 controls) by Du et al. (2014) suggested that the XPD 751Gln/Gln genotype was a low-penetrate risk factor for developing digestive tract cancers, especially in Asian populations.

## Conclusion

Since XPD is one of the major molecules which connect the two essential processes of sustenance of life–NER pathway and transcription process, it is one of the most analyzed molecules of NER in various epidemiological studies carried out on CRC. However, even though a decade of research on XPD gene and its SNPs, no clear relationships between its various SNPs and the risk of CRC has been established till date. To establish a cohesive data on XPD SNPs well-designed studies with large statistical power is warranted to clarify the ambiguity associated with the current data on XPD SNPs.

## Author contributions

All authors listed have made a substantial, direct and intellectual contribution to the work, and approved it for publication.

### Conflict of interest statement

The authors declare that the research was conducted in the absence of any commercial or financial relationships that could be construed as a potential conflict of interest.
